# ^11^C-Methionine PET for Identification of Pediatric High-Grade Glioma Recurrence

**DOI:** 10.2967/jnumed.120.261891

**Published:** 2022-05

**Authors:** Asim K. Bag, Melissa N. Wing, Noah D. Sabin, Scott N. Hwang, Gregory T. Armstrong, Yuanyuan Han, Yimei Li, Scott E. Snyder, Giles W. Robinson, Ibrahim Qaddoumi, Alberto Broniscer, John T. Lucas, Barry L. Shulkin

**Affiliations:** 1 Department of Diagnostic Imaging, St. Jude Children’s Research Hospital, Memphis, Tennessee;; 2 Department of Epidemiology and Cancer Control, St. Jude Children’s Research Hospital, Memphis, Tennessee;; 3 Department of Biostatistics, St. Jude Children’s Research Hospital, Memphis, Tennessee;; 4 Department of Oncology, St. Jude Children’s Research Hospital, Memphis, Tennessee;; 5 Department of Global Pediatric Medicine, St. Jude Children’s Research Hospital, Memphis, Tennessee; and; 6 Department of Radiation Oncology, St. Jude Children’s Research Hospital, Memphis, Tennessee

**Keywords:** MRI, ^11^C-MET PET, ^11^C-methionine PET, pediatric high-grade glioma, pseudoprogression, recurrence

## Abstract

Differentiating tumor recurrence or progression from pseudoprogression during surveillance of pediatric high-grade gliomas (PHGGs) using MRI, the primary imaging modality for evaluation of brain tumors, can be challenging. The aim of this study was to evaluate whether ^11^C-methionine PET, a molecular imaging technique that detects functionally active tumors, is useful for further evaluating MRI changes concerning for tumor recurrence during routine surveillance. **Methods:** Using ^11^C-methionine PET during follow-up visits, we evaluated 27 lesions in 26 patients with new or worsening MRI abnormalities for whom tumor recurrence was of concern. We performed quantitative and qualitative assessments of both ^11^C-methionine PET and MRI data to predict the presence of tumor recurrence. Further, to assess for an association with overall survival (OS), we plotted the time from development of the imaging changes against survival. **Results:** Qualitative evaluation of ^11^C-methionine PET achieved 100% sensitivity, 60% specificity, and 93% accuracy to correctly predict the presence of tumors in 27 new or worsening MRI abnormalities. Qualitative MRI evaluation achieved sensitivity ranging from 86% to 95%, specificity ranging from 40% to 60%, and accuracy ranging from 85% to 89%. The interobserver agreement for ^11^C-methionine PET assessment was 100%, whereas the interobserver agreement was only 50% for MRI (*P* < 0.01). Quantitative MRI and ^11^C-methionine PET evaluation using receiver-operating characteristics demonstrated higher specificity (80%) than did qualitative evaluations (40%–60%). Postcontrast enhancement volume, metabolic tumor volume, tumor-to-brain ratio, and presence of tumor as determined by consensus MRI assessment were inversely associated with OS. **Conclusion:** ^11^C-methionine PET has slightly higher sensitivity and accuracy for correctly predicting tumor recurrence, with excellent interobserver agreement, than does MRI. Quantitative ^11^C-methionine PET can also predict OS. These findings suggest that ^11^C-methionine PET can be useful for further evaluation of MRI changes during surveillance of previously treated PHGGs.

## Visual Abstract

**Figure:**
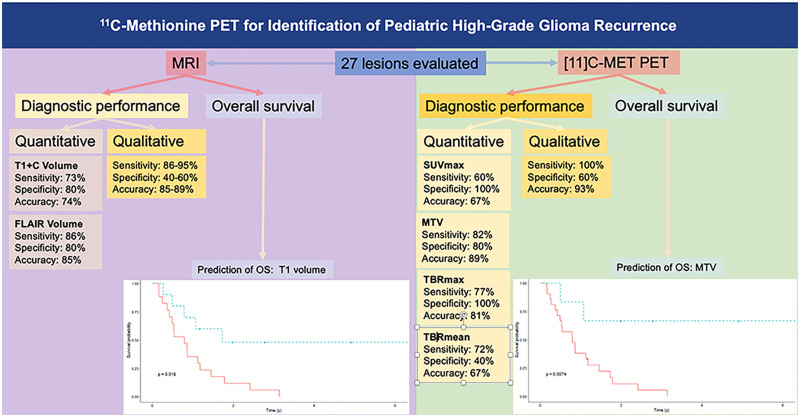

It has only recently been discovered that pediatric high-grade gliomas (PHGGs) are biologically distinct from adult high-grade gliomas (*1*). However, this new knowledge has not yet changed diagnoses, classifications, World Health Organization grading, or treatment of PHGGs (*2*). PHGGs in children older than 3 y are treated with a combination of maximal safe surgical resection, radiation therapy with or without adjuvant chemotherapy, and subsequent continued chemotherapy, similar to the treatment regimen for adult high-grade gliomas (*3*–*5*). Despite this aggressive therapy, outcomes in young children are dismal, with a local 1-y failure-free survival rate of 60% (*6*), suggesting that recurrence is common. Accurate diagnosis of tumor recurrence is important because the median overall survival (OS) of recurrent PHGGs is 4–7 mo (*7*) and because treatment of pseudoprogression is different from that of tumor recurrence. However, the diagnosis of recurrence is not always straightforward with MRI, which is the clinical standard-of-care test for assessing response to treatment. Indeed, treatment-related effects, including pseudoprogression, frequently mimic tumor recurrence, thereby leading to misdiagnosis and incorrect management (*8**,**9*).

Pseudoprogression is characterized by temporary enlargement and increased enhancement of clinical target volumes with MRI (*10*) and occurs in up to 20% of patients treated with radiation therapy and adjuvant chemotherapy (*11*). The incidence of pseudoprogression after initial therapy of PHGGs is similar to the incidence in adults after treatment of high-grade gliomas (*12*). Tumor recurrence is also characterized by enlargement of tumor volume, with increased enhancement making the distinction challenging (*13*–*15*). Many advanced MRI techniques have been extensively studied to differentiate treatment-related effects from true tumor progression, with variable benefits (*16*–*19*). PET with various radiotracers has been studied to distinguish true tumor progression from pseudoprogression (*17**,**20*–*24*). Of the many PET radiotracers used to evaluate tumor recurrence, study results using amino acid PET tracers (i.e., ^11^C-methionine, *O*-(2-^18^F-fluoroethyl)-l-tyrosine [^18^F-FET], and ^18^F-dihydroxyphenylalanine) in adults suggest that a reduction in amino acid uptake or a decrease in the metabolically active tumor volume is a sign of treatment response associated with long-term outcome (*25*). The Response Assessment in Neuro-Oncology working group and the European Association for Neuro-Oncology now suggest that ^18^F-FET may facilitate the diagnosis of pseudoprogression in glioblastoma patients within the first 12 wk after completion of chemoradiotherapy (*25*). ^11^C-methionine, a true amino acid PET tracer with properties similar to ^18^F-FET PET, has recently been shown to differentiate true tumor progression from treatment-related effects better than other PET tracers can in adults, with a sensitivity and specificity of 91.2% and 87.5%, respectively (*26*). Although the utility of ^11^C-methionine PET for evaluating nonenhancing PHGGs has been investigated (*27*), its use to evaluate tumor recurrence in PHGGs has not been systematically investigated.

Here, we evaluated whether ^11^C-methionine PET can be useful for the identification of tumor recurrence in previously treated PHGGs. Specifically, we compared the accuracy of ^11^C-methionine PET with that of MRI for predicting the presence of tumors when recurrence is suspected. We also compared the interobserver agreement of ^11^C-methionine PET and MRI to determine whether ^11^C-methionine PET imaging adds value to conventional MRI and whether ^11^C-methionine PET or MRI can predict OS.

## MATERIALS AND METHODS

### Study Subjects

We retrospectively included all subjects with PHGGs who were enrolled in the ongoing “Methionine PET/CT Studies in Patients with Cancer” clinical trial (NCT00840047) at St. Jude Children’s Research Hospital since 2009. This study was approved by the St. Jude Institutional Review Board, and each subject or a parent or legal guardian gave written informed consent to participate. The inclusion criteria for this study were as follows: previously treated World Health Organization grade III or IV PHGGs that demonstrated worsening or new imaging abnormalities on fluid-attenuated inversion recovery (FLAIR) sequences, on postcontrast T1-weighted sequences, or on both sequences during routine surveillance MRIs, in comparison with the MRI findings from the baseline or from the best response; ^11^C-methionine PET scans obtained within 3 wk of the surveillance MRI scans; and establishment of a definitive diagnosis of tumor recurrence within 8 wk of either the MRI surveillance scan or the ^11^C-methionine PET scan.

### Imaging Acquisition

#### ^11^C-Methionine PET

^11^C-methionine was prepared as previously described (*28*). ^11^C-methionine PET imaging followed at least 4 h of fasting. Each subject received intravenous injections of 740 MBq (20 mCi) of ^11^C-methionine per 1.7 m^2^ of body surface area (maximum prescribed dose, 740 MBq). Transmission CT images (for attenuation correction and lesion localization) and PET images were acquired approximately 5–15 min (mean ± SD, 8.7 ± 3.3 min) after ^11^C-methionine injection with a Discovery 690 PET/CT scanner or a Discovery LS PET/CT scanner (GE Healthcare) using these parameters: field of view, 30 cm; matrix, 192 × 192; reconstruction method, VUE point HD; quantification method, SharpIR; filter cutoff, 5.0 mm; subsets, 34; iterations, 4; and *z*-axis filter, standard. The Q.Clear 350 SharpIR quantification method was used in only 1 subject. The CT acquisition parameters were as follows: 0.5-cm slice thickness, 0.8-s tube rotation, 1.5 cm/rotation table speed, 1.5:1 pitch, 120 kV, and 90 mA with dose modulation. PET images were acquired in 3-dimensional mode for 15 min. Data were reconstructed into multiplanar cross-sectional images with standard vendor-supplied software and displayed on a nuclear medicine workstation (Hermes Medical Systems, Inc.) for analysis.

#### MRI

The following sequences were acquired with a 1.5-T Avanto magnet or a 3-T TrioTim, Skyra, or Prisma magnet (Siemens Medical Solutions) with a 0.1 mmol/kg dose of intravenous gadobutrol (Gadavist; Bayer Healthcare): 3-dimensional magnetization-prepared rapid gradient-echo (1 mm^3^ isotropic acquisition, 1,590-ms repetition time, 2.7-ms echo time, 900-ms inversion time, and 15° flip angle); 2-dimensional (2D) transverse T1-weighted fast low-angle shot (4-mm slice thickness, no gap, 259-ms repetition time, 2.46-ms echo time, and 70° flip angle); 2D transverse diffusion-weighted sequence and postcontrast 2D transverse T1-weighted fast low-angle shot (parameters identical to those of precontrast axial 2D T1-weighted); 2D transverse T2-weighted turbo spin-echo (4-mm slice thickness, no gap, 4,810-ms repetition time, 87-ms echo time, and 180° flip angle); 2D transverse T2-weighted FLAIR (4-mm slice thickness, no gap, 10,000-ms repetition time, 106-ms echo time, 2,600-ms inversion time, and 130° flip angle); and 3-dimensional sagittal T1-weighted (parameters identical to those of precontrast sagittal 3-dimensional T1-weighted). Apparent diffusion coefficient maps were calculated from the diffusion images with the vendor-provided software (Syngo; Siemens Healthcare).

### Qualitative Image Analysis

#### MRI

Each surveillance MRI was evaluated 4 times. The first evaluation was performed during generation of the clinical report by one of the neuroradiologists assigned to the clinical service. The second evaluation was performed by a single neuroradiologist (observer 1) with 12 y of experience evaluating response assessments in pediatric brain tumors. The third evaluation was performed by a single neuroradiologist (observer 2) with 8 y of experience evaluating response assessments in pediatric brain tumors. Both observers were masked to the ^11^C-methionine PET findings and did not have access to any clinical information or any imaging studies obtained after the index surveillance MRI. The fourth evaluation consisted of a consensus evaluation by observers 1 and 2. New or worsening MRI abnormalities were subjectively categorized as definitely tumor (score of 1), definitely not tumor (score of 2), or indeterminate (score of 3). The consensus readings were also scored with the same 1–3 scale. If a discrepancy in opinion occurred between 2 observers, the reading was scored as 3. The first rating from neuroradiologists on clinical duties was scored with the same scale on the basis of the clinical reports. Diffusion and apparent diffusion coefficient maps were used together for subjective evaluation only.

#### ^11^C-Methionine PET

^11^C-methionine PET images were independently reviewed by 2 observers, one with 15 y of experience and the other with 2 y of experience in molecular imaging for assessment of treatment response in pediatric brain tumors. The observers were provided the location of the MRI abnormality and had access to the MR images. The ^11^C-methionine PET images were rated qualitatively on a 4-point scale relative to frontal white matter (in all included subjects, at least some component of the frontal lobe white matter was free of tumor): 0, no detectable uptake; 1, mild uptake but less than in the contralateral frontal lobe white matter; 2, mild uptake similar to that in the contralateral frontal lobe white matter; or 3, uptake greater than in the contralateral frontal lobe white matter. Finally, the results of visual assessment were consolidated into just 2 groups. The first group was “no uptake or uptake the same as or lower than in the reference region” (grades 0, 1, and 2), and the second group was “uptake higher than in the reference region” (grade 3).

### Quantitative Imaging Analysis

Worsening or new imaging abnormalities on postcontrast T2-weighted FLAIR and T1-weighted sequences were manually segmented using Vitrea Advanced Visualization (Vital Images) software. Three patients had subtle enhancement on T1-weighted sequences, and their T1-weighted regions of interest were drawn on the Δ-T1 images (precontrast T1-weighted images were subtracted on a voxel-by-voxel basis from the postcontrast T1-weighted images).

SUVs for the ^11^C-methionine PET images were calculated using Hermes software. After coregistration of the PET dataset with FLAIR or postcontrast T1-weighted MRI sequences, regions of interest were manually drawn either around the areas of abnormal ^11^C-methionine uptake or around the MRI abnormality. In addition, quantitative tumor metrices (metabolic tumor volume and tumor-to-brain ratio [TBR]) were calculated as suggested by Law et al. (*29*). However, instead of using a crescentic region of interest, we used a 1.0-cm^3^ sphere to calculate the SUV_mean_ of the contralateral normal prefrontal lobe cortex and juxtacortical white matter as suggested by Hotta et al. (*22*) for consistency. Briefly, SUV_mean_ of the contralateral normal frontal lobe cortex and juxtacortical white matter was calculated using a 1.0 cm^3^ sphere. The 3-dimensional metabolic tumor volume with an SUV more than 1.3 times that of the normal brain cortex (obtained in the prior step) was automatically contoured using Hermes software, which automatically calculated the SUV_max_ and SUV_mean_ of the tumor. TBR and TBR_max_ were then manually calculated by dividing the tumor SUV_max_ by the SUV_mean_ of the contralateral normal frontal lobe cortex. TBR_mean_ was manually calculated by dividing the tumor SUV_mean_ by the SUV_mean_ of the contralateral normal frontal lobe cortex. In lesions with an SUV less than 1.3 times that of the contralateral frontal lobe, a volume of interest was manually drawn on the FLAIR-abnormal areas and agreed on by both nuclear medicine physicians, and then the volumes of interest were copied to the PET images. The SUV_max_ of the volumes of interest were automatically calculated by the software. The TBR was then calculated as described above.

### Final Outcomes

The final outcomes of the lesions evaluated with MRI and ^11^C-methionine PET were determined with the following methods: Response Assessment in Neuro-Oncology criteria applied to imaging and clinical findings (*30*); biopsies; or follow-up imaging and clinical course. Tumor was defined as present in the evaluated lesions if the lesions were treated as progressive disease (defined by Response Assessment in Neuro-Oncology criteria), if a predominant tumor was evident via biopsy, if progressive worsening was evident by follow-up MRI within 8 wk of the surveillance MRI or ^11^C-methionine PET scan, or if the subject died of tumor progression without any other identifiable cause. Because all evaluated lesions were included at recurrence, OS was calculated from the date of diagnosis of recurrent tumor or pseudoprogression.

### Statistical Analysis

MRI and ^11^C-methionine PET readings were defined as true positive when tumor scores correctly identified the final outcome and as false positives when tumors scores differed from the final outcome. Ratings were defined as true negatives when tumor scores did not correctly identify the final outcome and as false negatives when tumor scores did not differ from the final outcome. Sensitivity and specificity were calculated by standard statistical definitions. Accuracy was defined as the proportion of true positives and true negatives in all scans. Interobserver agreement between different MRI and ^11^C-methionine PET observers was calculated with Cohen κ-values, which were interpreted as previously indicated (*31*). Log-rank tests were used to assess the association of subjective ^11^C-methionine PET and MRI findings with OS. By using optimal cutoffs, we generated Kaplan–Meier curves for MRI parameters (T1-enhancing volumes, FLAIR volumes), and a PET parameter (SUV_max_) to test whether these measurements from quantitative imaging analysis were associated with OS.

The sensitivity and specificity of metabolic tumor volume, TBR, T1-enhancing volume, FLAIR volume, and SUV_max_ using optimal cutoffs for predicting final outcomes were evaluated. We used the optimized cutoffs to categorize these imaging features, and log-rank tests were performed to test whether each of these features was associated with OS values, which were calculated from the time of the MRI and ^11^C-methionine PET scans to the death of the subjects or—for subjects still alive—to the date of the last follow-up. The 95% CIs for all diagnostic accuracy measures were calculated using bias-corrected bootstrap methods with resampling. All statistical analyses were done using R Statistical Software.

## RESULTS

We used May 2020 as the cutoff for our analysis and found 27 patients who matched our inclusion criteria. We excluded 1 patient with l-2-hydroxyglutaric aciduria because differentiating tumor tissue from healthy brain was challenging because of diffuse brain signal abnormalities in the entire brain due to this condition. Of the remaining 26 patients, 27 tumors (1 patient had a left frontal lobe recurrence that was treated and evaluated similarly to the original tumor in the cerebellum) were included in the analysis. Details of patient demographics and tumors are shown in Table 1 and Supplemental Table 1 (supplemental materials are available at http://jnm.snmjournals.org). The details of the previous treatment, tumor location, and genetic alterations are included in Supplemental Table 2.

**TABLE 1.** Demographics of Patients Included in Study (*n* = 27)

Characteristic	Patients (*n*)
Diagnosis	
Glioblastoma	17
World Health Organization grade III astrocytoma	5
High-grade neuroepithelial tumor	2
High-grade glioma	2
Anaplastic pleomorphic xanthoastrocytoma	1
Age at time of PET imaging (y)	
0–5	4
6–10	2
11–15	8
16–20	8
20–25	4
Sex	
Male	16
Female	10
Patient status	
Deceased	22
Alive	4

**TABLE 2.** Diagnostic Accuracy for Tumor Detection

Index	Qualitative MRI reading	Qualitative PET reading	T1-enhancing volume	FLAIR volume	SUV_max_	MTV	TBR_max_	TBR_mean_
Sensitivity	0.95 [0.71–1]	1 [NA]	0.73 [0.50–0.88]	0.86 [0.64–0.96]	0.60 [0.36–0.78]	0.90 [0.69–1]	0.77 [0.55–0.91]	0.72 [0.50–0.88]
Specificity	0.60 [0–1]	0.60 [0–1]	0.80 [0–1]	0.80 [0–1]	1 [NA]	0.80 [0–1]	1 [NA]	0.40 [0–1]
Accuracy	0.89 [0.67–0.93]	0.93 [0.7–0.96]	0.74 [0.52–0.85]	0.85 [0.63–0.93]	0.67 [0.44–0.81]	0.89 [0.64–0.96]	0.81 [0.59–0.89]	0.67 [0.44–0.78]

PET = ^11^C-methionine PET; MTV = metabolic tumor volume; NA = not applicable.

Data in brackets are 95% CIs.

### Qualitative MRI and ^11^C-Methionine PET Interpretations for Predicting Final Outcomes

The final outcome in 5 of the 27 lesions evaluated were no tumor present (i.e., pseudoprogression), and in the remaining 22 lesions it was presence of tumor (i.e., tumor progression). The final outcomes were confirmed by follow-up MRI in 16 cases, by biopsy in 4, and by Response Assessment in Neuro-Oncology criteria in 7.

The sensitivity, specificity, and accuracy of correctly predicting the presence of tumors from MRI were 86% (95% CI, 64%–96%), 80% (95% CI, 0%–100%), and 85% (95% CI, 63%–93%), respectively, for observer 1 and 95% (95% CI, 73%–100%), 40% (95% CI, 0%–100%), and 85% (95% CI, 63%–93%), respectively, for observer 2. The interobserver agreement was fair (Cohen κ = 0.49; *P* < 0.001). The sensitivity, specificity, and accuracy for correctly predicting the presence of tumors by consensus readings were 95% (95% CI, 71%–100%), 60% (95% CI, 0%–100%), and 89% (95% CI, 67%–93%), respectively. The details are summarized in Table 2.

**FIGURE 1.**
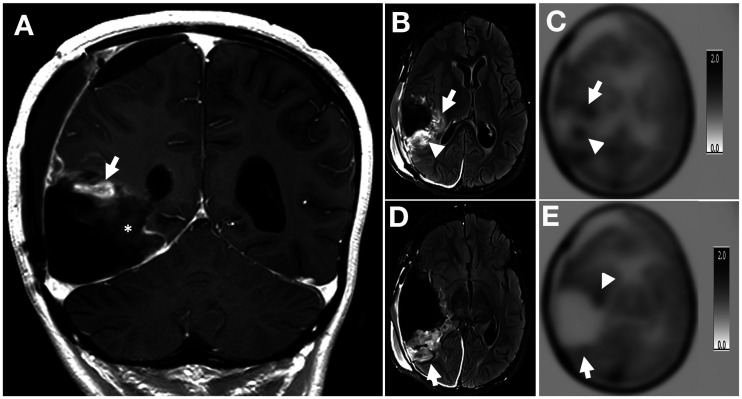
(A) Postcontrast coronal T1-weighted image demonstrates nodular enhancement (arrow) at superior surgical margin. (B) Axial T2-weighted FLAIR image obtained through level of nodular enhancement seen in A demonstrates areas of heterogeneously hyperintense tissue at medial (arrow) and posterior (arrowhead) surgical margin. (C) Axial reconstruction of ^11^C-methionine PET images through this level shows 2 foci of tracer uptake at medial (arrowhead) and posterior (arrow) surgical margin. (D) Axial T2-weighted FLAIR image obtained through plane (demarcated by asterisk in A) inferior to plane of images B and C demonstrates relatively large areas of heterogeneously hyperintense tissue at posterior surgical margin (arrow). (E) Axial reconstruction of ^11^C-methionine PET images through this level shows no ^11^C-methionine uptake at posterior surgical margin (arrow). There is minimum uptake at anteromedial surgical margin (arrowhead). This area was not included in metabolic tumor volume because of low SUV (lower than 1.3 times that of contralateral frontal lobe cortex).

The sensitivity, specificity, and accuracy for correctly predicting the presence of tumors with ^11^C-methionine PET scans were 100% (95% CI, not applicable), 60% (95% CI, 0%–100%), and 93% (95% CI, 70%–96%), respectively, and the interobserver agreement was 100% (Cohen κ = 1). Positive ^11^C-methionine PET readings had higher sensitivity, specificity, and accuracy for correctly predicting the presence of tumors than did individual MRI readings. ^11^C-methionine PET also had higher sensitivity and accuracy for correctly predicting the presence of tumors than did the consensus MRI readings. The consensus MRI and ^11^C-methionine PET readings were concordant in 88.9% of cases and discordant in 11.1%. In 1 subject, there was significant discrepancy between the MRI abnormality and the PET abnormality; in this subject, there were considerable surgery-related MRI abnormalities because the scans were obtained 21 d after surgery (Fig. 1).

**FIGURE 2.**
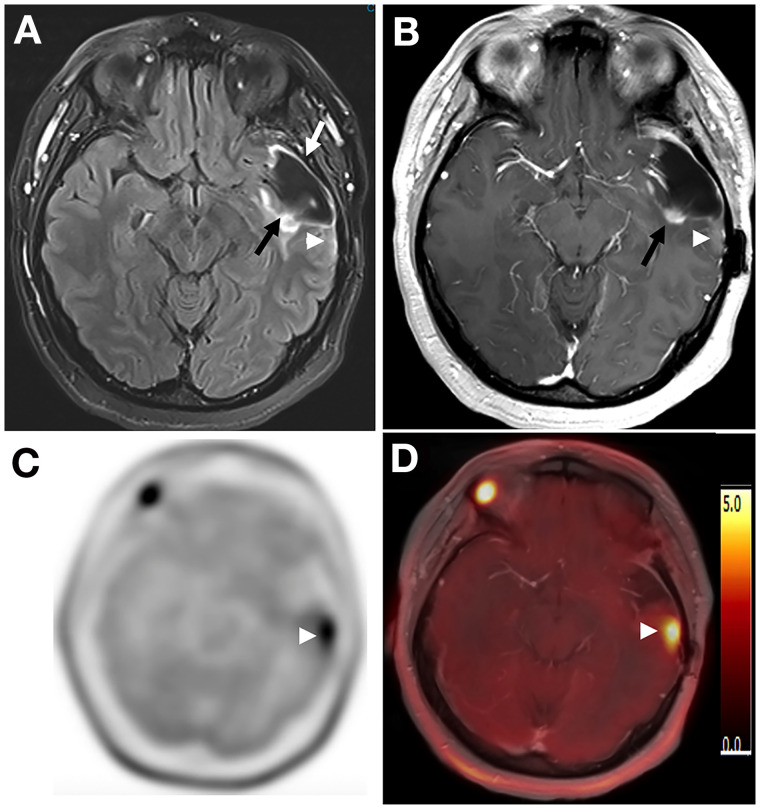
(A) Axial T2-weighted FLAIR image through level of midbrain shows large cystic resection cavity in left temporal lobe (white arrow). There is ill-defined T2 abnormality at medial aspect of resection cavity (black arrow). No obvious abnormality is noted posterior and lateral to resection cavity (arrowhead). (B) Axial postcontrast T1-weighted image through same level better shows focal area of contrast enhancement (arrow). This enhancing focus has been followed up since prior treatment. Subtle contrast enhancement, new finding compared with previous MRIs, is noted posterior and lateral to resection cavity (arrowhead). (C) Axial reconstruction of ^11^C-methionine PET images through same level shows intense ^11^C-methionine uptake posterior and lateral to resection cavity (arrowhead) corresponding to new subtle T1 enhancement. (D) Postcontrast T1-weighted ^11^C-methionine PET/MRI image also shows that ^11^C-methionine abnormality corresponds to new subtle enhancement at posterior and lateral aspect of resection cavity (arrowhead).

We tested the accuracy between MRI observer 1, MRI observer 2, MRI consensus reads, and ^11^C-methionine PET reads in pairs with McNemar tests. There were no significant differences for any pair in the comparisons. In 5 of the 27 lesions, a discrepancy occurred between MRI observer 1, MRI observer 2, or the consensus MRI read for correctly predicting the final outcome, but ^11^C-methionine PET correctly predicted the final outcomes in all these cases. The final outcome of 3 of these 5 lesions was presence of tumor, and the final outcome of 2 of these lesions was pseudoprogression. Only 1 case was indecisive for changes related to tumor treatment versus changes not related to tumor treatment in the consensus MRI interpretation but was correctly predicted by the ^11^C-methionine PET evaluation (Fig. 2).

### Quantitative Imaging Parameters from Both ^11^C-Methionine PET and MRI for Predicting Final Outcomes

The receiver-operating-characteristic curves for SUV_max_, metabolic tumor volume, TBR_max_, TBR_mean_, T1-enhancing tumor volume, and abnormal tumor volume by FLAIR were assessed for their ability to predict the final outcomes (*32*). The optimal SUV_max_ cutoff to differentiate between the presence and absence of tumors was 3.3, with sensitivity, specificity, and accuracy of 60% (95% CI, 36%–78%), 100% (95% CI, not applicable), and 67% (95% CI, 44%–81%), respectively. The optimal metabolic tumor volume cutoff was 0.98 cm^3^, with sensitivity, specificity, and accuracy of 90% (95% CI, 69%–100%), 80% (95% CI, 0%–100%), and 89% (95% CI, 64%–96%), respectively. The optimal TBR_max_ cutoff was 1.82, with sensitivity, specificity, and accuracy of 77% (95% CI, 55%–91%), 100% (95% CI, not applicable), and 81% (95% CI, 59%–89%), respectively. The optimal TBR_mean_ cutoff was 1.4, with sensitivity, specificity, and accuracy of 72% (95% CI, 50%–88%), 40% (95% CI, 0%–100%), and 67% (95% CI, 44%–78%), respectively. The optimal T1-enhancing volume cutoff was 2.4 cm^3^ or greater, with sensitivity, specificity, and accuracy of 73% (95% CI, 50%–88%), 80% (95% CI, 0%–100%), and 74% (95% CI, 52%, 85%), respectively. The optimal abnormal FLAIR volume cutoff was 13.76 cm^3^, with sensitivity, specificity, and accuracy of 86% (95% CI, 64%–96%), 80% (95% CI, 0%–100%), and 85% (95% CI, 63%–93%), respectively. The details are summarized in Table 2.

### Quantitative MRI and ^11^C-Methionine PET Interpretations Associated with OS

**FIGURE 3.**
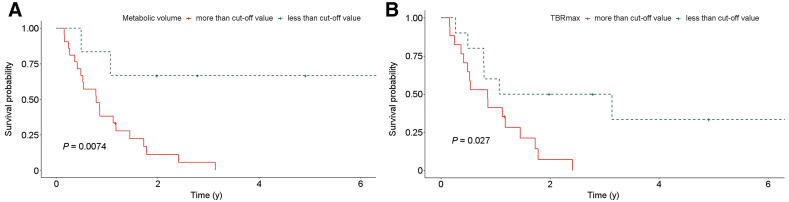
Kaplan–Meier curves demonstrating OS probability of subjects according to ^11^C-methionine PET quantitative metrics. *P* values of log-rank tests of Kaplan–Meier curves are given for metabolic tumor volume (A) and TBR_max_ (B).

**FIGURE 4.**
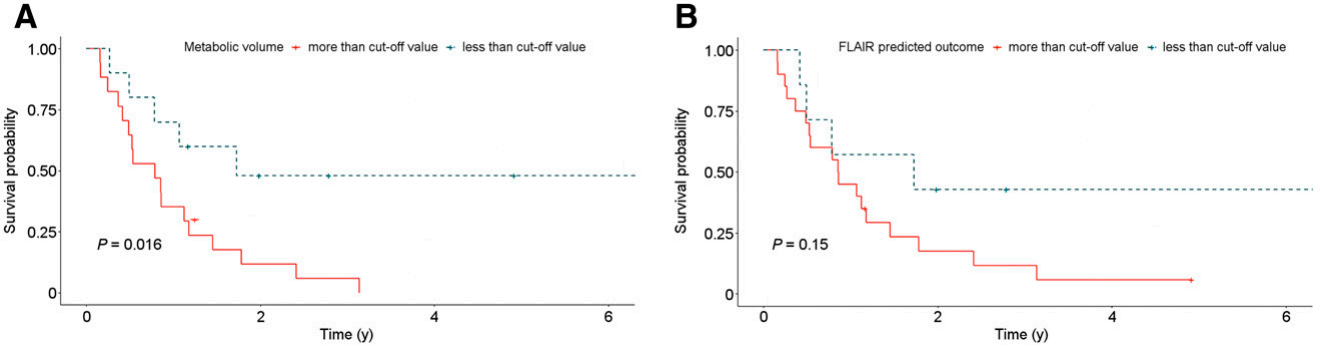
Kaplan–Meier curves demonstrating OS probability of subjects according to quantitative MRI metrics. *P* values of log-rank tests of Kaplan–Meier curves are given for postcontrast T1-enhancing volume (A) and FLAIR volume (B).

We used the optimized cutoffs to categorize imaging features, including the T1-enhancing volume, FLAIR volume, SUV_max_, metabolic tumor volume, and TBR. T1-enhancing tumor volume, metabolic tumor volume, and TBR were significant by themselves for predicting the final outcome. However, the association of final outcome with quantitative imaging parameters was not significant when tested with multivariable analysis. Log-rank tests were performed to test whether these imaging features are associated with OS. Using the cutoffs determined by receiver-operating-characteristic curves, we found that OS was significantly associated with metabolic tumor volume (*P* = 0.0074), TBR_max_ (*P* = 0.027), and T1-enhancing volume (*P* = 0.016) (Figs. 3 and 4). However, SUV_max_, TBR_mean_, and FLAIR volume did not show a significant association with OS.

## DISCUSSION

Differentiating true tumor progression from treatment-related effects can be challenging because of overlapping features (*11**,**19**,**25*). Many advanced MRI techniques and molecular imaging techniques have been studied to address this challenge (*19**,**25*). Recent evidence suggests that amino acid PET tracers (i.e., ^18^F-dihydroxyphenylalanine PET and ^18^F-FET PET) can assist conventional MRI at correctly identifying surgical margins and distinguishing between tumoral and nontumoral changes (*15**,**33*–*36*). ^11^C-methionine PET, in particular, has shown substantial promise (*37*–*40*), but these studies were performed only on adults, and many included metastatic nonprimary CNS tumors. Therefore, we explored the role of ^11^C-methionine PET in evaluating only recurrent PHGGs.

The ^11^C-methionine uptake is directly related to L-type amino acid transporter 1 expression (*41*); high ^11^C-methionine uptake characteristically occurs in tumors with a high degree of neoangiogenesis and cellular proliferation (*8**,**41*). Previous studies have found ^11^C-methionine PET to have high sensitivity and specificity for diagnosing high-grade tumors (*8**,**42*). In our study, we found that the sensitivity and accuracy of ^11^C-methionine PET for correctly differentiating true tumor progression from treatment-related effects were 100% and 93%, respectively, compared with the reported 70%–80% sensitivity and 75% accuracy in previous studies (*37**,**38**,**40*). This difference may be due to the heterogeneous samples in the previous studies, which included both metastases and gliomas that were treated with different radiation doses and chemotherapy regimens. However, the sensitivity and specificity of the ^11^C-methionine PET for differentiating tumor progression from treatment-related effects in our study were similar to the results of a study by Dunkl et al. (*43*). Our study also found the quantitative PET evaluation to have higher specificity than qualitative evaluation. This is in contrast to a study by Minamimoto et al. (*37*), which found no significant difference between qualitative and quantitative ^11^C-methionine PET evaluations for assessment of tumor progression. More recently, a study by Marner et al. also found ^18^F-FET PET to have high specificity and accuracy for differentiating tumor from nontumor lesions (*44*).

Qualitative interpretation of MRI findings is the standard of care for follow-up of high-grade gliomas after treatment (*19*). Unlike qualitative ^11^C-methionine PET assessments, qualitative interpretation of MRI findings involves careful evaluation of many different MRI sequences that exploit the different magnetic properties of tissues and changes in these magnetic properties with MRI contrast compounds. This multifactorial evaluation process inherently leads to interpretation bias, as we observed in our study. The sensitivity, specificity, and accuracy of the 2 MRI observers in our study significantly differed, although both observers had expertise in evaluating pediatric brain tumors for 10 y or more. Such interpretation bias influences the diagnostic performance of MRI; indeed, we found that the consensus MRI interpretation performed significantly better, similar to that of ^11^C-methionine PET, than did the individual MRI readings. Because consensus MRI interpretations by multiple neuroradiologists are not practical in routine clinical practice, the addition of ^11^C-methionine PET imaging for suggestive MRI findings adds value to the overall care of patients with PHGGs.

Our study demonstrated a significant association of metabolic tumor volume and TBR_max_ with OS, as previously described (*45**,**46*). Additionally, postcontrast T1-enhancing volume was also significantly associated with OS, similar to multiple prior studies (*47**,**48*).

Our study included limitations. The sample size was small but relatively large, considering the rarity of this tumor. As this study was initiated in 2009, the acquisition time of our PET scan was set to 15 min instead of the currently recommended 20 min. In addition, the criteria for performing ^11^C-methionine PET on the included patients were based on a high clinical suspicion for recurrence or a high likelihood of tumor recurrence on MRI findings. Consequently, there was a high pretest probability that the MRI abnormalities would represent tumor recurrence, thereby introducing selection bias. A larger prospective multiinstitutional study with regularly scheduled ^11^C-methionine PET scans might alleviate such selection bias. These studies should be sufficiently powered to examine whether ^11^C-methionine PET SUV_max_ cutoffs and qualitative interpretations can quantitatively predict final outcomes. However, because of the short half-life of ^11^C (∼20 min), ^11^C-methionine is currently available only at institutions with access to a cyclotron; such a study would need to be restricted to centers with ^11^C-methionine–synthesizing capability or institutions able to refer patients with suggestive findings on MRI to a center with ^11^C-methionine–synthesizing capability. To mitigate this problem, ^18^F-FET PET with a longer half-life is increasingly used in assessments of gliomas in many countries (*49*–*52*).

## CONCLUSION

Our study showed that ^11^C-methionine PET has slightly higher sensitivity, specificity, and accuracy for correctly predicting the presence of tumor recurrence than does MRI when new or worsening imaging abnormalities are detected during surveillance of previously treated PHGG. The interobserver agreement on interpretation for ^11^C-methionine PET findings was excellent and better than that of MRI. Our study also showed that quantitative ^11^C-methionine PET and MRI can also predict OS. These findings indicate that ^11^C-methionine PET imaging may add value for predicting PHGG recurrence. However, the results from this small cohort should be validated in larger prospective, preferably multiinstitutional studies.
